# On the track of transfer cell formation by specialized plant-parasitic nematodes

**DOI:** 10.3389/fpls.2014.00160

**Published:** 2014-05-05

**Authors:** Natalia Rodiuc, Paulo Vieira, Mohamed Youssef Banora, Janice de Almeida Engler

**Affiliations:** ^1^Laboratório de Interação Molecular Planta-Praga, Embrapa Recursos Genéticos e Biotecnologia, PqEBBrasília, Brasil; ^2^NemaLab – Instituto de Ciências Agrárias e Ambientais Mediterrânicas, Universidade de ÉvoraÉvora, Portugal; ^3^Department of Plant Pathology, Faculty of Agriculture, Ain Shams UniversityCairo, Egypt; ^4^Institut National de la Recherche Agronomique, Plant, Health and Environment, Plant-Nematodes Interaction Team, UMR 1355 ISA/Centre National de la Recherche Scientifique, UMR 7254 ISA/Université de Nice-Sophia Antipolis, UMR ISASophia-Antipolis, France

**Keywords:** nematode feeding sites, transfer cells, wall ingrowths, galls, syncytia, root-knot nematodes, cyst nematodes

## Abstract

Transfer cells are ubiquitous plant cells that play an important role in plant development as well as in responses to biotic and abiotic stresses. They are highly specialized and differentiated cells playing a central role in the acquisition, distribution and exchange of nutrients. Their unique structural traits are characterized by augmented ingrowths of invaginated secondary wall material, unsheathed by an amplified area of plasma membrane enriched in a suite of solute transporters. Similar morphological features can be perceived in vascular root feeding cells induced by sedentary plant-parasitic nematodes, such as root-knot and cyst nematodes, in a wide range of plant hosts. Despite their close phylogenetic relationship, these obligatory biotrophic plant pathogens engage different approaches when reprogramming root cells into giant cells or syncytia, respectively. Both nematode feeding-cells types will serve as the main source of nutrients until the end of the nematode life cycle. In both cases, these nematodes are able to remarkably maneuver and reprogram plant host cells. In this review we will discuss the structure, function and formation of these specialized multinucleate cells that act as nutrient transfer cells accumulating and synthesizing components needed for survival and successful offspring of plant-parasitic nematodes. Plant cells with transfer-like functions are also a renowned subject of interest involving still poorly understood molecular and cellular transport processes.

## INTRODUCTION

The plant cell wall consists of a dynamic extracellular complex that responds to external and internal cellular signals, and forms a bridge between the plasma membrane and the cytoskeleton (Humphrey et al., 2007). The cell wall is formed of a network of polysaccharides and proteins and is multifunctional in plants: it maintains and determines the cell shape (Szymanski, 2009; Singh and Montgomery, 2011), resists internal turgor pressure (Haswell et al., 2008), controls cell and plant growth (Wolf et al., 2012), contributes to plant morphology (Hamant et al., 2010), regulates diffusion through the apoplast and is involved in perception and signaling during plant development and defense mechanisms (Hamann, 2012; Nühse, 2012; Underwood, 2012). Plant cell walls are composed of primary and secondary walls. The primary cell wall is laid down during cytokinesis and keeps expanding until cells acquire their final shape. The composition and heterogeneity of cell walls rely on developmental programs, in addition to environmental conditions (Burton et al., 2010). Secondary cell walls are thicker, and are deposited at the inner side of the primary cell wall mainly in highly specialized tissues and cell types such as xylem vessels and fiber cells. While most cells deposit a uniformly thickened secondary wall, some cells, e.g., tracheary elements (Hogetsu, 1991) and transfer cells (TCs; Gunning and Pate, 1974), build up an intricate secondary wall at restricted regions. TCs are highly specialized cells that are found in algae and fungi, and in all *taxa* of the plant kingdom, suggesting that every plant has the genomic ability to develop TCs under a particular array of environmental status and/or developmental signals (Gunning and Pate, 1974; Offler et al., 2003; Andriunas et al., 2013). TCs are situated at regions of functional nutrient transport (Gunning and Pate, 1969, 1974) with the multifaceted wall ingrowth/plasma membrane complex often oriented to the track of solute flow. They facilitate apo/symplastic exchange of solutes and their cytoplasm is typically dense and organelle rich, with numerous mitochondria and organelles of the endomembrane secretory system situated nearby the extended wall ingrowths (Gunning et al., 1968; Davis et al., 1990). Vacuoles in TCs may be small or not present.

Generally, TCs develop from a range of differentiated cell types by a process that involves de-differentiation followed by re-differentiation named *trans*-differentiation (Andriunas et al., 2013). Examples are xylem or phloem parenchyma cells, pericycle and epidermal cells. Since TCs arise from differentiated plant cells, these are named according to the initial cell type, e.g., companion-cell TCs (Gunning et al., 1968; Wimmers and Turgeon, 1991; Haritatos et al., 2000), nucellar projection TCs (Wang et al., 1994), and so on. The *trans*-differentiation process occurs either during the normal developmental course of a particular plant tissue or takes place in response to an abiotic or biotic stress. The ensuing TC has a distinctive wall harboring intricately invaginated ingrowths unsheathed by a plasma membrane enriched in nutrient transporter proteins (Offler et al., 2003). Ingrowths on walls in TCs generally present the reticulate or flange architecture or a combination of both (Talbot et al., 2002). TCs may well develop at both sides of the tissue interface or only at one side and ingrowths may be asymmetrically distributed.

Although little is known about the molecular signals that induce TC differentiation, some genes expressed associated with TCs have been described (Hueros et al., 1995, 1999; Gómez et al., 2002; Gutiérrez-Marcos et al., 2004; Muñiz et al., 2006). Among these the Myb-related protein-1 (*MRP-1*) was the first TC-specific transcriptional activator identified in plants (Gómez et al., 2002) and was shown to be a key regulator of TCs differentiation process in maize endosperm (Gómez et al., 2009). In addition, MRP-1 regulates the expression of several TC-specific genes, like *BETL-1* and *BETL-2* (Gómez et al., 2002), *Meg-1* (for *Maternally Expressed Gene 1*; Gutiérrez-Marcos et al., 2004), and *TCRR-1* (for transfer cell response regulator 1; Muñiz et al., 2006), through its interaction with the corresponding promoters (Barrero et al., 2006) and of *BETL-9* and *BETL-10* promoters (Gómez et al., 2009).

Transfer cells can also develop associated with biotic symbionts (nitrogen-fixing bacteria and mycorrhiza) and plant pathogens (e.g., nematodes, leafhoppers, fungus; Pate and Gunning, 1972; Offler et al., 2003). TC establishment is also linked to interactions connected with a reciprocally beneficial trade of nutrients between host and symbiont. Examples are *Frankia* hyphae on *Alnus rubra* root hair infection directing the development of nitrogen-fixing root nodules (Berry et al., 1986), or root epidermal cells in association with mycorrhizas (Allaway et al., 1985) and *Rhizobium* nodules on pea roots (Gunning et al., 1968). Examples of TC induction in response to pathogen strike comprise injury of leafhopper on companion cells of *Medicago sativa* (alfalfa) internodes (Ecale-Zhou and Backus, 1999) and disease caused on *Duchesnea indica* leaf cells by rust fungus (Mims et al., 2001).

**FIGURE 1:**
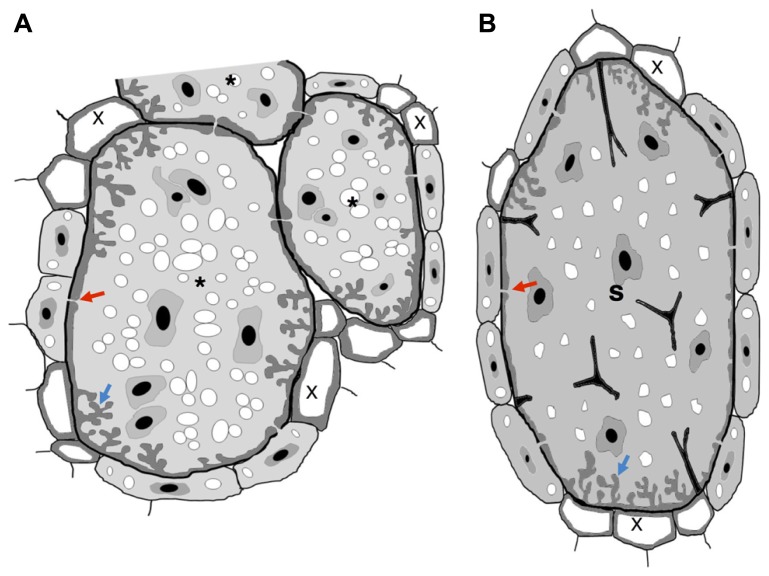
**Schematic view of nematode feeding transfer-cells induced by plant-parasitic nematodes. (A)** Giant cells induced by RKN show cell wall thickenings with invaginations (blue arrow) often at the proximity of xylem vessels. Plasmodesmata (red arrow) also connect giant cells with phloem cells to facilitate solute transfer and may connect NCs. **(B)** Syncytium induced by a CN show cell wall thickenings with invaginations (blue arrow) often at the proximity of xylem vessels. Plasmodesmata (red arrow) also connect a syncytium with phloem cells to facilitate solute transfer and may connect NCs. Wall stubs are the result of cell dissolution of several root cells that fused to the syncytium itself. Asterisk, giant cell; X, xylem; S, syncytium.

Infection of plant roots by plant-parasitic nematodes also lead to the development of root swellings containing specialized host-derived feeding structures, with which nematodes acquire nutrients. The most studied specialized feeding sites are induced by root-knot (RKN, *Meloidogyne* spp.) and cyst (CN, *Globodera* spp., *Heterodera* spp.) nematodes, designated giant cells and syncytia, respectively (Jones and Northcote, 1972a,b). However, other minor economic species belonging to other *taxa*, such as *Rotylenchulus* spp., *Nacobbus* spp., and *Xiphinema* spp., are also able to induce specialized feeding sites in the host roots. In the case of RKN and CN, both feeding-cell types have the function to feed the pathogen (Jones and Northcote, 1972a,b; Schemes in **Figures 1A,B**). Products secreted by nematodes through their stylet induce the differentiation of root cells into feeding structures and the content of this secretion remains largely unidentified (Mitchum et al., 2013).

The molecular and cellular processes involved in solute transport in plant tissues via TCs is yet poorly understood, even though vital for the survival of plants and particular biotrophic plant pathogens. This review will focus on data available on cells with transfer-like function induced by biotrophic sedentary plant-parasitic nematodes, such as RKN and CN nematodes. Cytological similarities between TCs suggest that at least part of the nematode feeding site developmental pathway might involve common routes regulating TC morphology and function.

## NEMATODE INDUCED TRANSFER CELLS: CELLULAR REARRANGEMENTS AND FUNCTION

Nematodes are devastating plant pathogens that trigger yield losses in numerous crop plants. A great part of the damage is caused by sedentary nematodes, which induce specialized feeding sites in plant roots, from which nutrients are withdrawn. Amongst these pathogens, CN (family Hoplolaimidae) and RKN nematodes (family Meloidogynidae) are considered the major economically important plant parasitic species (de Almeida Engler et al., 2005). Feeding sites induced by CN and RKN are regarded as resilient metabolic sinks (Grundler and Hofmann, 2011; Bartlem et al., 2014). Even though both feeding systems share common structural and functional features, their ontogeny differs considerably.

**FIGURE 2:**
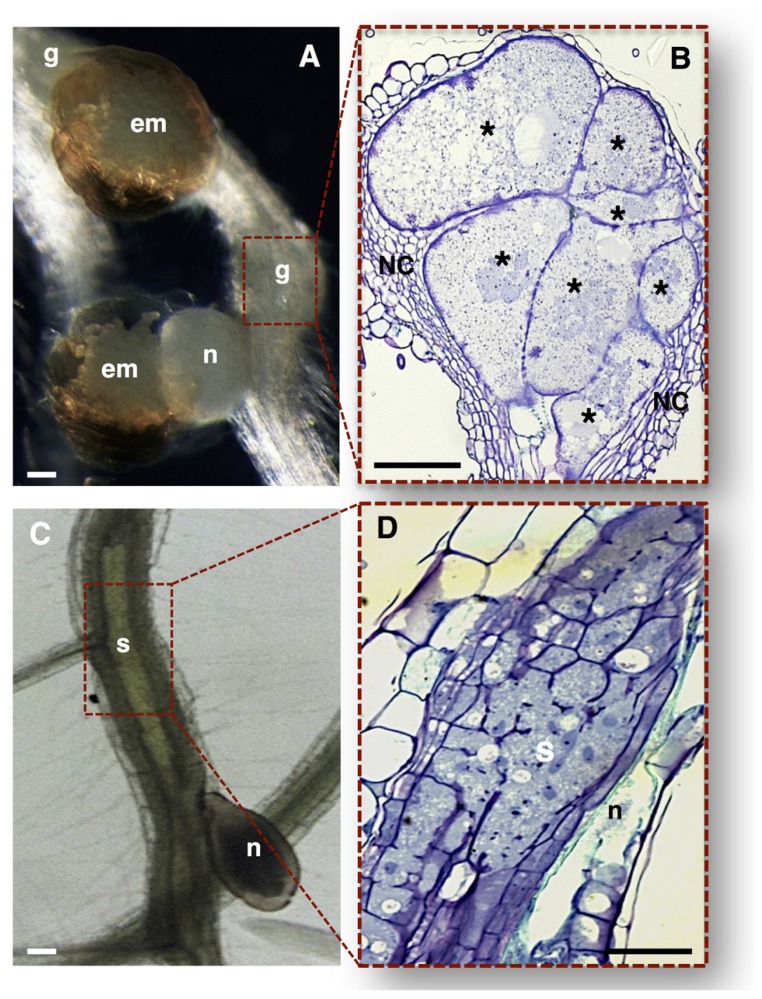
**Nematode feeding sites induced by specialized sedentary biotrophic plant-parasitic nematodes. (A,B)** *Arabidopsis thaliana* roots infected with the RKN *Meloidogyne incognita.* **(A)** A typical gall induced in the plant host containing a mature female nematode, and associated gelatinous matrix filled with nematode eggs. **(B)** Longitudinal gall section containing seven multinucleate giant cells with unevenly thickened cell walls, surrounded by asymmetrically divided NCs. **(C,D)** *Arabidopsis thaliana* roots infected with the CN *Heterodera schachtii.* **(C)** A typical syncytium induced in plant host roots associated with a female CN. **(D)** Detailed longitudinal section of a syncytium resulting from the cell wall dissolution of several root cells. em, egg masses; g, gall; n, nematode; NC, neighboring cells; Asterisk, giant cells; S, syncytium. Bars = 50 μm.

Root-knot nematodes induce galls composed of giant cells surrounded by neighboring cells (NCs), giving the root a shape of a knot (**Figures 2A,B**; de Almeida Engler et al., 1999, 2010). Giant cells are generated through sequential mitoses without cytokinesis (Huang and Maggenti, 1969; de Almeida Engler et al., 2004) and cycles of DNA replication (Jones and Payne, 1978; Wiggers et al., 1990; de Almeida Engler et al., 1999, 2012), leading to nuclear and cellular hypertrophy.

Cyst nematodes induce syncytia, formed by an initial feeding cell followed by fusion of hundreds of NCs causing root distension (**Figures 2C,D**; de Almeida Engler et al., 1999, 2010; Sobczak et al., 2011). The multinucleated state in a syncytium is most probably attained by cell wall dissolution of NCs (Endo, 1978) rather than by mitotic activity. Increase in cytoplasmic density and nuclear volume (de Almeida Engler et al., 1999, 2012) and cell wall modifications (Jones and Goto, 2011; Sobczak and Golinowski, 2011) have been observed for both syncytia and giant cells. Both feeding sites lead to root swellings disturbing water and nutrient transport thereby affecting plant growth.

Different arguments have been attributed to the choice of nematodes to induce feeding cells at the vascular parenchyma. The position of the initial syncytial cell ensures close contact of the feeding site at the proximity of the xylem and phloem necessary to provide nutrients to the developing gall (Bartlem et al., 2014) or syncytium. As well, the selected cells of the vascular tissue may be more amenable to the nematode-induced changes. Vascular parenchymal cells are not entirely differentiated and may thus be arrested at a particular cell cycle phase, allowing the switch to other cell types like the nematode feeding site (de Almeida Engler et al., 2011). Roles of cortical or endodermal cells outside the vascular cylinder tissue have not yet been ascribed to syncytia induced by the CN, *Heterodera schachtii*, nor to galls induced by the RKN, *Meloidogyne incognita*, in *Arabidopsis thaliana* roots.

**FIGURE 3:**
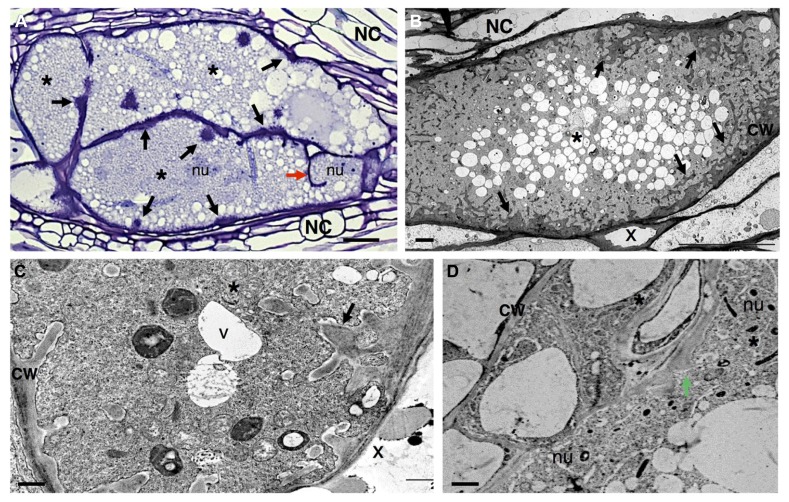
**Anatomy of *Meloidogyne incognita-*induced giant cells in *Arabidopsis thaliana* roots. (A)** Light microscopy image of sectioned giant cells embedded in a gall and stained with toluidine blue. Cell wall thickenings (black arrows), and a cell wall stub (red arrow) indicating arrest of cytokinesis. **(B–D)** Ultra-structure of giant cell sections showing cell wall ingrowths (black arrows) along regions predominantly flanking the vascular tissue. Note the xylem elements with thickened cell walls and dense cytoplasm containing numerous organelles including asymmetrically shaped nuclei and small vacuoles. **(D)** Detailed giant cells showing a PD (green arrow). Asterisk, giant cell; NC, neighboring cells; x, xylem; CW, cell wall; V, vacuole; nu, nucleus. Bars = **(A)** 25 μm and **(B–D)** 5 μm.

**FIGURE 4:**
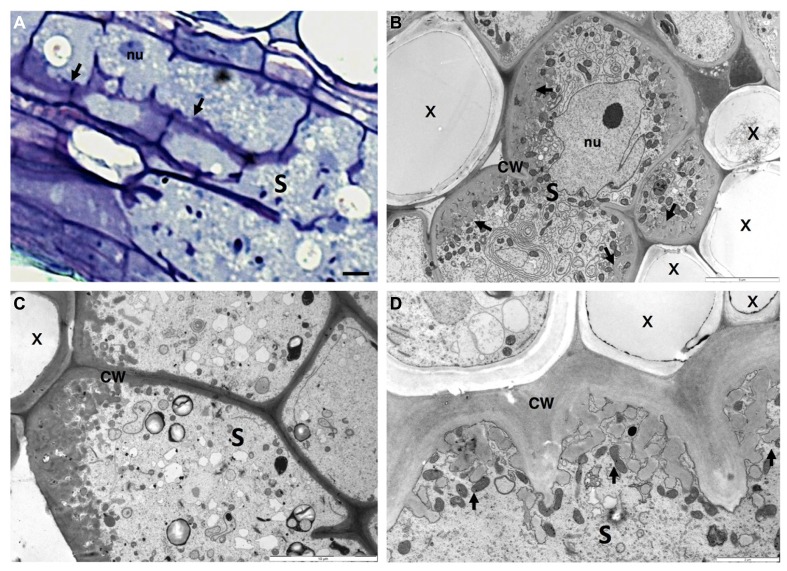
**Anatomy of *Heterodera schachtii* induced-syncytium in *Arabidopsis thaliana* root cells. (A)** Light microscopy of a maturing syncytium section stained with toluidine blue, presenting cell wall thickenings (black arrows). **(B–D)** Ultra-structure of syncytia sections showing cell wall ingrowths along regions mainly flanking the vascular tissue. Note the xylem elements with thickened cell walls and the dense cytoplasm containing numerous organelles including asymmetrically shaped nuclei and small vacuoles. S, syncytium; x, xylem; CW, cell wall; nu, nucleus. Bars = **(A)** 25 μm.

In the past years there has been extensive data reporting on the anatomy of the sophisticated nematode feeding sites induced by CN and RKN, comprising light, scanning and transmission electron microscopy (e.g., Bird, 1961; Jones and Dropkin, 1976; Wergin and Orion, 1981; Hussey and Mims, 1991; Berg et al., 2008; Sobczak and Golinowski, 2008). Both nematode feeding sites share common features, such as the increase of metabolic activity and cytoplasmic density, the replacement of a large central vacuole by several smaller ones, the large nuclei number of increased size, and the proliferation of organelles including Golgi stacks, mitochondria, plastids, ribosomes, and endoplasmic reticulum (**Figure 3**; Vieira et al., 2013 and **Figure 4**: Berg et al., 2008; Sobczak et al., 2011). Concomitant with the structural modifications in a gall or a syncytium, cell walls thicken and finger-like protuberances (ingrowths or cell wall labyrinths) form (Schemes in **Figures 1, 3** and **4**; Berg et al., 2008; Sobczak et al., 2011; Vieira et al., 2013) with the function to increase the membrane surface area for solute uptake (e.g., Golinowski et al., 1996; Hussey and Grundler, 1998). The cell wall degradation is also observed in syncytia (Scheme in **Figures 1B** and **2D**; de Almeida Engler et al., 1999 and **Figures 4A,B**; Sobczak et al., 2011). Extensive changes of cell wall architecture in diverse types of TCs encountered in plants may comprise cell wall ingrowths and partial cell wall degradation (Offler et al., 2003) as occurring in syncytia. Increased giant cell wall ingrowths accompanied by intense surrounding vascularization will certainly contribute to the access to nutrient supply by the feeding nematode (Bartlem et al., 2014). Similar intense vascularization around syncytia will certainly enhance nutrient supply to the developing nematode.

Modifications of plant cell walls within nematode feeding cells appear to be coordinated by significant changes in host gene expression, as highlighted by a range of methods (e.g., Gheysen and Fenoll, 2002; Jammes et al., 2005; Hammes et al., 2005; Ithal et al., 2007a,b; Gheysen and Mitchum, 2009; Barcala et al., 2010; Damiani et al., 2012). Examples are plant cell wall and metabolism genes, shown to be differentially expressed in nematode feeding sites compared to uninfected root tissue. Although the origin of thickened cell walls, and elaborate cell wall labyrinths of combined reticulate or flange architecture are still not well understood, the elaborate structural design of nematode feeding cell walls reflects the hyperactivity of the cell wall synthesis machinery of the host plant. Similarly to walls of plant TCs (Offler et al., 2003), feeding cells are mainly composed of polysaccharides such as cellulose, hemicelluloses and pectin (Dropkin and Nelson, 1960; Littrell, 1966).

The large repertoire of host genes encoding plant cell wall modifying enzymes distinctively regulated in giant cells include for example; an extensin (*EXT*, Niebel et al., 1993), the expansin gene family (several members of α- and β-*expansins*; Bar-Or et al., 2005; Jammes et al., 2005; Gal et al., 2006), a pectin acetylesterase (putative pectin acetylesterase, *PAE* homologue; Vercauteren et al., 2002), pectate lyases (*PEL*; Jammes et al., 2005) and endoglucanases (*endo-β-1,4-glucanases*; Goellner et al., 2001; Sukno et al., 2006).

Similar cell wall related genes are up-regulated in syncytia, such as expansins (α- and β*-expansins*; Golecki et al., 2002; Wieczorek et al., 2006; Fudali et al., 2008; Griesser and Grundler, 2008), endoglucanases (*endo-β-1,4-glucanases*; Goellner et al., 2001; Wieczorek et al., 2008), *EXT,* and extension-like (*EXTL*) genes (Ithal et al., 2007a,b); polygalacturonases (*PG*; Mahalingam et al., 1999) and pectin acetylases (*PE*; Vercauteren et al., 2002; Ithal et al., 2007a,b). This data is suggestive of both unique and apparently common mechanisms orchestrated by these nematodes to provoke adaptations of the plant cell wall to facilitate feeding cell expansion and function.

Apart from cell wall changes pathogens may also locally interfere with signaling pathways changing the concentration of sugars such as sucrose (Hofmann et al., 2007) or plant hormones (e.g., Goverse and Bird, 2011; Rodiuc et al., 2012) in a coordinated manner that may directly or indirectly influence the function of feeding cells to act as TCs. Nematode secretions are likely to contain the proteins or peptides that can affect plant gene expression (Davis et al., 2008). In addition, for successful parasitism, nematodes can make use of plant genes as observed for the plant cell cycle or cytoskeleton machinery (de Almeida Engler et al., 1999, 2004, 2012; de Almeida Engler and Favery, 2011). Thus, nematodes may manipulate genes involved in cell wall rearrangements inducing the* trans*-differentiation of parenchyma vascular cells into giant or syncytial TCs.

## CELL WALL MODIFICATIONS AND CELLULAR COMMUNICATION IN ROOT-KNOT NEMATODE-INDUCED GIANT CELLS

As mentioned above, RKN species induce root galls containing giant-feeding cells in a large variety of plant hosts. In giant cells induced by RKN, wall thickening is observed as patches expanding, merging, branching and often of uneven cell wall material deposition at early stages of giant cell development suggesting an existing mechanism responsible for depositing irregular cell wall material (**Figure 3**; de Almeida Engler et al., 2004; Mordechai and Oka, 2006; Berg et al., 2008). Patches of wall thickenings are distributed in giant cells mainly in the proximity of the proliferating phloem and xylem elements, involved in the transfer of water and solutes, and may include NCs (Jones and Northcote, 1972a; Hoth et al., 2008). Throughout giant cell maturation these cell wall patches expand and cover large areas, generating regions with profuse reticulate TC wall labyrinths (Scheme in **Figures 1A** and **3**; Berg et al., 2008; Vieira et al., 2013). It is fascinating to observe that cell wall ingrowths proliferate as RKN develop, then degenerate as nematodes reach maturity and complete their life cycle. Cell wall composition of young giant cells is similar to syncytia. Thus, these progressive cell wall changes suggest that the molecular dialog between nematodes and plant hosts is continuous, and might hold a key role in maintenance of the physiological status of giant cells (Jones and Northcote, 1972a).

Giant cells also present cell wall fragments, or stubs, thought initially to originate from cell wall breakdown as was observed in syncytia (Jones and Dropkin, 1975; **Figure 3A**; Vieira et al., 2013). Subsequently, it has been demonstrated that cell wall stubs are the product of the abortion of phragmoplast expansion and cell wall formation (Jones and Payne, 1978; de Almeida Engler et al., 2004).

**FIGURE 5:**
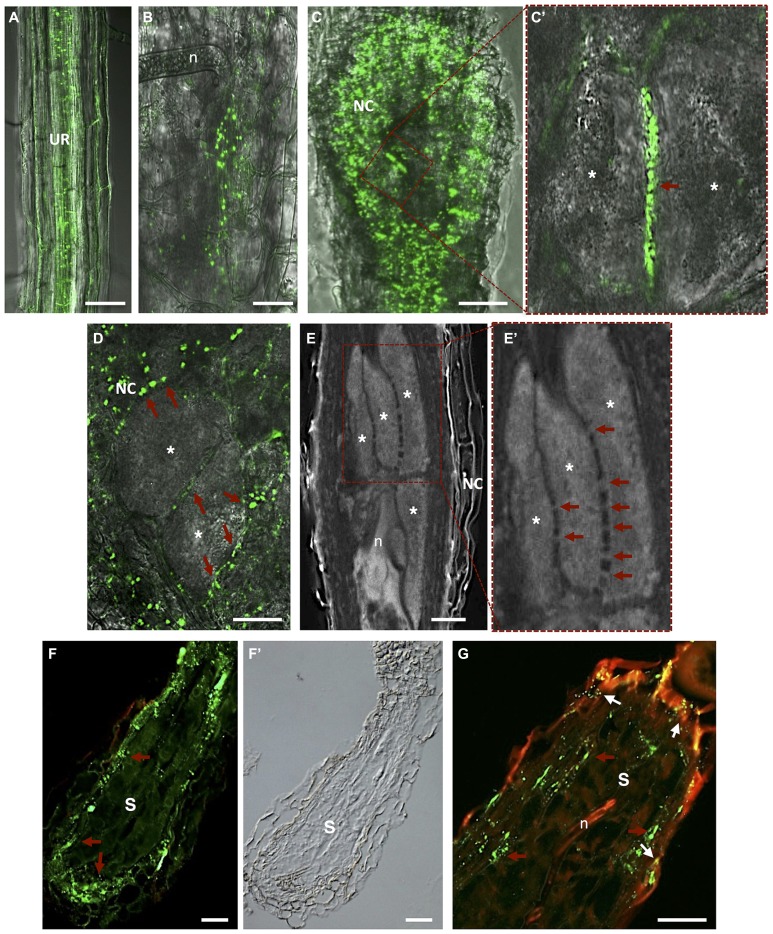
**Plasmodesmata localization in *Meloidogyne incognita*-induced galls and *Heterodera schachtii-*syncytium in *Arabidopsis thaliana* roots. (A)** *In vivo* localization of MP17^PLRV^-GFP (plasmodesmata localization marked by green fluorescence). In an uninfected root; **(B)** in a gall at early stage after nematode infection; and **(C)** in a mature gall. **(C’)** Detail of two adjacent giant cells containing numerous PD (red arrow). **(D)** *In vivo* MP17^PLRV^-GFP localization between two giant cells and connecting NCs. Observations of Figures **A–D** were made on non- and infected material of *Arabidopsis* transgenic lines (35S:MP17^PLRV^-GFP). Non-infected roots, and galls were dissected from roots, embedded in 5% agar and fresh slices were observed using an inverted confocal microscope. **(E)** Cleared whole-mount gall showing the complex network of PD between giant cells. **(E’)** Detail of giant cells containing numerous PD (red arrows). **(F,G)** PD (red arrows point to green fluorescence of PD) in a section of a syncytium flanked by NCs, and **(F’)** a differential interference contrast image is presented to show syncytium tissue morphology. **(G)** Double localization of PD (red arrows point to green fluorescence of PD) and callose (white arrows to yellow dots). Green dots hint at open PD whereas yellow dots suggest that solute transport can be blocked by callose in syncytia. UR, uninfected root; n, nematode; NC, neighboring cells; Asterisk, giant cell; S, syncytium. Bars = 50 μm.

A significant demand for nutrients from feeding cells is created by nematodes. This is manifested by the development of TC wall labyrinths of wall ingrowths, an idea long sustained as a hallmark of giant cells (Jones and Northcote, 1972a,b; Jones and Gunning, 1976). These wall ingrowths notably increase the surface area of the plasma membrane, assisting the transport of nutrients into or out of the feeding cell, i.e., like symplast–apoplast exchange occurring in plant TCs (Gunning and Pate, 1969; Gunning et al., 1974; Offler et al., 2003). Furthermore, TC wall labyrinths can be observed on the cell walls of neighboring giant cells, indicating that nutrient transport in the apoplast, pooled from outlying cells, can be an important source of giant-cell nutrients. As shown by Berg et al. (2008), walls lying between giant cells are thickened and labyrinth-rich, suggesting that nutrients might also flow between these feeding cells (Jones and Northcote, 1972b; Jones and Gunning, 1976). As well, solutes that are phloem-derived are imported into the giant cells either via plasmodesmata (PD) (symplastically; **Figure 3D**; Vieira et al., 2013 and **Figures 5B–E**’; Hofmann et al., 2010; Vieira et al., 2012) or by means of active transport (apoplasmically).

Hofmann et al. (2010) gave insights into the role of PD frequency and distribution and callose deposition along cell walls of giant-feeding cells. This survey employed the double localization of callose and green fluorescent protein (GFP) in root sections. Initially, giant cells were reported to be symplastically isolated from NCs (Jones and Dropkin, 1976; Wolf et al., 1991; Hoth et al., 2008). Further studies using* Arabidopsis* transgenic plants containing a viral movement protein (MP) of the *Potato leaf roll polerovirus* fused to GFP (MP17^PLRV^-GFP; Hofius et al., 2001) as a PD marker (**Figure 5A**; Roberts and Oparka, 2003; Hofmann et al., 2010), reported that giant cells are connected by PD (Hofmann et al., 2010). No callose deposition has been detected in galls except in a number of NCs (Hofmann et al., 2010). Most viral MP attach to branched PD that are essentially secondary PD formed in existing cell walls. Primary PD are often not branched and appear during cell wall formation after cell division.

Plasmodesmata are unique membrane channels in plant cell walls that provide cytoplasmic continuity, cell-to-cell transport, and an intercellular exchange network (Crawford and Zambryski, 2000). The status of PD may change from closed to opened, allowing the flow of small or larger metabolites only when pertinent for plant tissue development (Kim and Zambryski, 2005). Inward compression of the plasma membrane can also play a role in reducing the size exclusion limit of the PD, or eventually cutting off solute passage (Wolf et al., 1991). As well, passage of most molecules is controlled by the size exclusion limit and therefore macromolecules such as proteins and RNA rely on specific trafficking processes. Passage through PD has to be tightly regulated due to its importance in signaling for information and solute exchange during plant cell development. In plant–nematode interactions, callose has been detected around the nematode stylet inserted into plant cells of *Criconemella xenoplax* (Hussey et al., 1992) and *H. schachtii* (Sobczak et al., 1999) and along PD of syncytia (Jones and Payne, 1977; Grundler et al., 1998). Deposition of callose (β-1,3-glucan) in the cell wall contiguous to PD, at both ends of the channel, may control the passage of water and solutes and can be transient or reversible. Numerous PD can be found in the cell walls of giant cells induced by RKN in *Arabidopsis*, suggesting massive symplastic solute transfer (**Figure 5D**; Hofmann et al., 2010). PD were detected not only in walls between giant cells but also in walls of NCs, including the proliferating vascular tissue (**Figures 5B–E**’; Hofmann et al., 2010; Vieira et al., 2012). Immunocytochemical analysis verified that these PD were not functionally impaired due to potential callose deposition, contrary to what is observed for syncytia. This suggests that the stress that RKN might cause to vascular parenchyma root cells dedifferentiated into feeding cells is not enough to induce callose deposition. The occurrence of cell wall ingrowths and PD at the feeding site induced by RKN thus imply that these cellular adaptations are responsible for bulk solute transport across the plasma membrane and via symplastic transport. In addition, solute transport may be aided by specialized membrane transport proteins that regulate the flow of nutrients into and out of giant cells. Giant cell morphology indicates that areas occupied by cell wall ingrowths have a lower frequency of PD (Huang and Maggenti, 1969; Jones, 1981) and thus regions with less wall ingrowths contain a higher density of PD.

## CELL WALL MODIFICATIONS AND CELLULAR COMMUNICATION IN A CYST NEMATODE-INDUCED SYNCYTIUM

The initial syncytial cell induced by CN originates from a procambial cell for *Heterodera* spp. or a cortical parenchyma or endodermis cell for *Globodera* spp. (Golinowski et al., 1996; Sobczak et al., 2005). The first visible changes in this initial syncytial cell include alterations to the plant cell wall configuration and cell wall dissolution (**Figures 2D** and **4B**; Golinowski et al., 1996; de Almeida Engler et al., 1999; Sobczak et al., 2011). The syncytium expands along the host root, NCs divide and fuse, and some cells differentiate into new xylem tissue (vessels) and phloem cells (sieve elements; Berg et al., 2008; Hoth et al., 2008). Although nematodes produce a range of cell wall degrading enzymes in their esophageal gland cells, which are secreted through the stylet (Mitchum et al., 2013), their specific involvement in the cell wall degradation within the syncytium is still unclear. The activity of plant cell wall degrading enzymes (Goellner et al., 2001) in syncytia suggest that degradation of cell walls in syncytium is mainly accomplished by plant enzymes, while nematode-specific enzymes assume greater importance for cell wall degradation and loosening during the migration of nematodes through the host root. Degradation of cell walls in syncytia accompanies the cell wall synthesis needed to produce cell wall ingrowths (Jones and Northcote, 1972a; Sobczak et al., 1997) close to the xylem and for the thickening of the outer cell wall of the syncytium (Golinowski et al., 1996).

In syncytia, finger-like cell wall ingrowths are elongated (Scheme in **Figures 1B** and **4**; Sobczak et al., 2011), branch and form sophisticated reticulate labyrinths that expand apically, causing the basal parts of ingrowths to fuse, developing into extensive cell wall thickenings. Deposition of wall ingrowths is only obvious around 5 to 7 days after CN infection once feeding cell development is well advanced (Golinowski et al., 1996). This indicates that wall ingrowth development may be a secondary response, unrelated to nematode feeding cell development (Jones and Northcote, 1972a), and might be caused by the augmented flow of solutes to the feeding nematode. While cell walls flanked by the syncytial elements are locally broken down, incorporating NCs into the syncytium (Golinowski et al., 1996; Grundler et al., 1998), the outer cell walls seem to be extended and thickened to resist augmented turgor pressure created inside the syncytium (Golinowski et al., 1996; Wieczorek et al., 2006). During nematode development, cell wall ingrowths fuse and form distinct depositions along the cell wall of the CN-induced syncytium. Thus, the formation and deposition of new ingrowths are continuously fashioned during nematode maturation (Golinowski et al., 1996; Sobczak et al., 2011), resulting in an increase of surface area of the plasma membrane at the interface predominantly between syncytium and xylem elements and phloem cells, facilitating water and nutrient transport into the syncytium.

Cell wall ingrowths occurring in syncytia, typical for TCs, involve myo-inositol oxygenases (MIOX; Kanter et al., 2005), which are strongly expressed in syncytia (Szakasits et al., 2009). *MIOX* genes are involved in the production of UDP- glucuronic acid, a precursor of sugars used for cell wall biosynthesis, and potentially involved in ascorbate synthesis (Lorence et al., 2004). Thus, studies covering different aspects of cell wall rearrangements in a CN induced-syncytium reveal adjustments in cell wall morphology, thickness and possibly composition, presumably essential to maintain a functional feeding site. Likewise, the presence of cell wall ingrowths with transfer-like function most likely plays an important role in nutrient translocation to allow nematode development and reproduction.

Openings caused by cell fusion in the forming syncytium may also develop from PD that are widened and subsequently enlarged by gradual dissolution of cell walls (Jones and Northcote, 1972a; Wyss et al., 1984; Grundler et al., 1998). During the maturation of a syncytium wall in contact with vascular cells, invaginations develop at regions in close contact with the secondary xylem and phloem elements (**Figure 4C**; Golinowski et al., 1996; Sobczak et al., 2011). Nematode feeding is highly depending on transfer of solutes from neighboring phloem and xylem elements. The cell wall of a young syncytium thickens uniformly except for walls in contact with sieve tubes, which remain thin until the moment neighboring sieve tube cell wall start thickening (Grundler et al., 1998). Thus, an established syncytium is surrounded by thickened walls where NCs continue to be incorporated by progressive and local cell wall dissolution (**Figures 4A,B**; Sobczak et al., 2011). New cell wall openings can also be created between NC walls with no involvement of PD. After dissolution of the cell wall and middle lamella the plasmalemma fuses and the protoplast of the NC is incorporated into the syncytium. Often remnants of cell walls are present within an expanding syncytia (**Figures 2D** and **4**; de Almeida Engler et al., 1999; Sobczak et al., 2011). Only a few PD are detected in young syncytia (**Figures 5F,F**’; Hofmann et al., 2010) and a temporal callose deposition implies impaired symplastic exchange (**Figure 5G**; Grundler et al., 1998; Hofmann et al., 2007, 2010). Co-localization of MP17^PLRV^-GFP and callose confirmed the isolation of syncytia during the first days of feeding site development (**Figure 5G**; Hofmann et al., 2010). In the meantime, the outer syncytial wall becomes thickened and newly deposited cell wall material obstructs existing PD. This will lead to the symplastic isolation of young syncytia, thus nutrients are transported from phloem apoplastically via transmembrane carriers (Juergensen et al., 2003; Hofmann and Grundler, 2006). During syncytium development (4–7 days after inoculation-DAI) an increased frequency of PD is observed with less callose deposition, confirming previous fluorochrome transport studies (Hofmann and Grundler, 2006; Hofmann et al., 2007). At later developmental stages (>10 DAI), microscopy studies, in addition to microinjection assays, confirmed that syncytia are symplastically connected to new host phloem assembled as sieve elements and companion cells as illustrated by the presence of PD (Hoth et al., 2005, 2008; Hofmann et al., 2007, 2010). This symplastic transport is essential for transfer of nutrients into the syncytia and for nematode development.

It is a widespread event that plant tissues can switch between symplastic isolation to connectivity during development and functional analysis can validate this status. This has been shown for *Arabidopsis* embryos (Kim and Zambryski, 2005), or during cotton fiber elongation characterized by a period of symplastic isolation followed by increased expression of plasma membrane transporters and decreased callose PD gating (Ruan et al., 2001, 2004). Yet when mature syncytia are not symplastically isolated sucrose transporters are still required for additional sugar retrieval.

Functional analysis of the T-DNA line with an insertion in the exon of the β-1,3-glucanase (*AtBG_ppap*; Levy et al., 2007) showed a reduced size of syncytia and the increased ratio of male nematodes after CN infection, suggesting stress conditions (Hofmann et al., 2010). The β-1,3-glucanase enzyme degrades callose deposited along PD. Decreased syncytial size most likely affected nutrient availability and nematode development, also affecting sexual differentiation (Betka et al., 1991). A second mutant line with a T-DNA insertion in the putative callose synthase gene *GLUCAN SYNTHASE-LIKE5* (*AtGSL5*), although showing a strong reduction in wound callose and papillary callose formation after mechanical wounding and infection with *Sphaerotheca fusca* (Jacobs et al., 2003), had no negative effect on nematode development (Hofmann et al., 2010). In fact, reduced callose deposition might facilitate PD-dependent cellular fusion during syncytium expansion, increasing its volume, thus enhancing nutrient availability for nematode growth (Hofmann et al., 2010).

Thus, the presence and functionality of PD in nematode-induced TCs may affect their structure, development, maintenance and morphogenesis through their impact on solute exchange. Unrestricted PD paths will assist intracellular communication facilitating solute import needed for the prompt nematode development and efficient reproduction.

## PLASMA MEMBRANE AND TRANSPORT FUNCTION OF NEMATODE FEEDING SITES

The main functions of TCs are nutrient and water transport. Both galls and syncytia are terminal sink tissues that require direct access to the plant vascular tissue (Jones, 1981; Grundler and Hofmann, 2011). For nematodes, a food source coming from their feeding-TCs is essential for their development and reproduction. The presence in TCs of the typical “wall-membrane apparatus” provides evidence of an efficient mechanism facilitating transmembrane transport of solutes. Cell wall ingrowths are surrounded by the plasmalemma, amplifying significantly the symplast–apoplast interface (Pate and Gunning, 1972; Golinowski et al., 1996; Offler et al., 2003). The plasmalemma is tightly linked to cell wall protusions and microtubules localized along wall ingrowths (Berg et al., 2008). Therefore, the large wall-membrane surface encountered in nematode feeding sites will facilitate great volumes of solute transfer. Sedentary nematodes need large amount of solutes containing nutrients and water to develop and reproduce in a short time. Thus, the presence of wall ingrowths increases short-distance solute transport between the apoplast (cell wall compartment) and symplast (cytoplasmic compartment) in plant cells. TCs are not the only cells particularly adapted for fast transport. As well, impressive solute fluxes can occur via PD symplastically linked as observed in nematode feeding sites (Hoth et al., 2008; Hofmann et al., 2010). Changes in cell size, as seen for giant cells, can also increase plasma membrane area as for cell wall ingrowths (Jones, 1981). As well, in species where TCs are absent, cells bordering the interface can become specialized for a transport function. Therefore, cells neighboring nematode feeding sites might be engaged to perform an analogous function. The occurrence of invaginations in giant cell walls and the large feeding-cell surface surrounded by NCs with transfer function would indubitably increase the efficiency of these feeding cells to import and translocate solutes to the feeding nematode. Increased plasma membrane surfaces in giant cells also requires increased proton pump and molecule carriers used for the translocation of nutriens to giant cells to nourish the nematode. As a consequence, genes encoding constitutive enzymes and structural proteins may be altered in their expression in order to support the increased cellular metabolic activity related to nematode feeding (Bleve-Zacheo and Melillo, 1997). Giant cells seem to employ a proton-coupled transport system located between the plasma membrane at wall ingrowths and xylem vessels (Dorhout et al., 1992; Grundler and Böckenhoff, 1997). Amino acid and sugar transport into plant cells is commonly assumed to be mediated by a proton force (Rheinhold and Kaplan, 1984) and there is evidence for a chemiosmotic model of proton-amino acid symport (Bush, 1993; DeWitt and Sussman, 1995). Sucrose/proton symport has also been observed in protoplasts derived from epidermal TCs of developing broad-bean cotyledons (McDonald et al., 1996).

Plasma membranes of TCs display large membrane potential dissimilarities (ranging from –150 to –200 mVat sites of solute influx; Jones et al., 1975; Robards and Stark, 1988; Bonnemain et al., 1991). These values are similar to other cell types implicated in solute influx like root hairs (Miedema et al., 2001). Membrane potential variation results from the activity of H+-ATPases, and most of these membrane proteins have been identified in plasma membranes of different TC types (Bouche-Pillon et al., 1994; McDonald et al., 1996; Harrington et al., 1997; Tegeder et al., 1999; Bagnall et al., 2000). The H+-ATPase gene has been found to be upregulated upon nematode infection in tomato roots (Bird and Wilson, 1994). So far, two members of the Ca^2^^+^-ATPase (*ACA*) family were verified to be upregulated in young nematode feeding sites (Hammes et al., 2005), implying a high energy demand (McClure, 1977; Gheysen and Fenoll, 2002). AtACA4 was localized to small vacuolar structures (Geisler et al., 2000) and is abundant at early stages of nematode feeding site development. Contrastingly, AtACA8 was located at the plasma membrane (Bonza et al., 2000) and its expression is higher in nematode feeding sites compared to uninfected root tissues, suggestive of playing a role during gall development. Thus, *AtACA4, AtACA8*, and *AtCAX3* (Hirschi, 2001) a member of the Ca^2^^+^:cation antiporter family, were proposed to modulate Ca^2+^-mediated signaling events in plant cells (Sanders et al., 1999; Manohar et al., 2011), and most likely in nematode-induced feeding cells. Data generated by these studies suggests that Ca^2^^+^ influx and signaling are involved in nematode-induced giant-TC development (Hammes et al., 2005).

As mentioned herein, nematode feeding sites have been described as sink tissues supplied with phloem-derived solutes such as sugars. Sucrose has been described as the main transported sugar in the phloem of *Arabidopsis* from source to sink tissues (Haritatos et al., 2000; Kühn, 2003) and phloem is loaded both apoplasmically and symplastically. Sucrose is also the major source of carbohydrate into nematode feeding sites and metabolite analyses revealed considerably augmented sucrose levels in nematode-induced syncytia and giant cells (Hofmann et al., 2007; Baldacci-Cresp et al., 2012). Similarly, Hofmann et al. (2008) proposed that syncytia as induced plant structures make use of starch as an intermediate carbohydrate storage to compensate for the fluctuating sugar levels taking place during nematode feeding and development.

Phloem-specific sucrose transporters seem also to be involved in solute transport in nematode feeding sites. AtSUC2 is thought to recover sucrose into the phloem (Truernit and Sauer, 1995; Weise et al., 2000) whilst AtSUC4 seems to be in charge of phloem loading and unloading (Weise et al., 2000). In addition, sucrose transporters play a role in the development of sink tissues in various plant organs (Gottwald et al., 2000; Kühn, 2003). In young syncytia symplastic pathways are not functional and sucrose transporters like *AtSUC2* and *AtSUC4* seem critical for importing sucrose into syncytia (Juergensen et al., 2003; Hoth et al., 2008). Hofmann et al. (2007) reported that *AtSUC4* silencing affected nematode development, suggesting its role during early syncytium development when no functional PD were present. Thus, it is believed that transporters are responsible for sucrose supply in young syncytia whereas at a later stage, connection to the phloem is established via PD although transporters seem also required for sucrose retrieval (Hofmann et al., 2007). The need to use different stratagems for solute retrieval from syncytia is understandable, given that a mature feeding site expands along the host root and must supply the nematode with enough solutes to grow and reproduce. In giant cells, sucrose is a major osmolyte and *AtSUC1* induction is most likely involved in sucrose transport (Hammes et al., 2005).

Recently, Cabello et al. (2013) investigated the role of sucrose cleaving enzymes like sucrose synthases (SUS) and invertases (INV) during the CN, *H. schachtii* and RKN,* Meloidogyne javanica* development, using single and multiple *INV* and *SUS* mutants. Both genes were shown to be transcriptionally regulated in nematode feeding sites (Szakasits et al., 2009; Barcala et al., 2010). Elevated sugar pools in multiple *INV* and *SUS* mutant lines promoted nematode development suggesting that these sugars have important nutritional value for the nematodes that may cleave sucrose with their own INVs (Grundler et al., 1991; Cabello et al., 2013). Syncytia and the plant shoot apex within these mutants exhibited changed sugar levels and enzyme activity suggestive of changes in the source-sink movement (Trouverie et al., 2003; Cabello et al., 2013). For the RKN, *Meloidogyne javanica*, development within *INV* and *SUS* mutants presented similar effects as observed for CN (Cabello et al., 2013). Hofmann et al. (2009) investigated, by Affymetrix gene chip, the expression of all genes annotated as sugar transporters in syncytia compared to non-infected roots using the *Arabidopsis* Membrane Protein Library. Expression of three significantly up-regulated (*STP12, MEX1*, and *GTP2*) and three down-regulated (*SFP1, STP7,* and *STP4*) genes in syncytia were validated by quantitative RT-PCR. While *STP4, STP7,* and *STP12* belong to the STP sugar transport protein family, *MEX1* and *GTP2* are plastidial transporter genes, and *SFP1* is a senescence-related monosaccharide transporter. A T-DNA insertion line of the most up-regulated gene (*STP12*) showed that insufficient sugar in feeding sites resulted in increased male ratios since females need higher sugar concentration in order to grow and reproduce (Hofmann et al., 2009). In addition, fluorescent-labeled glucose and membrane potential recordings performed following the application of several sugars deciphered sugar transporter activity across the plasma membrane of syncytia (Hofmann et al., 2009). Analyses of soluble sugar pools demonstrated a typical composition for syncytia. Besides sucrose, previously reported by Hofmann et al. (2007), glucose, galactose, raffinose, fructose, and trehalose were detected. Thus, sugar transporters are expressed and active in syncytia, indicative of their role in inter- and intracellular transport processes.

Besides sugars, amino acids are important as nutrient supply for nematode growth and development. Amino acids are the main form of transported organics, which is reduced nitrogen in the majority of plant species. Upon RKN infection noteworthy changes in the expression of genes involved in amino acid transport have been detected (Hammes et al., 2005, 2006; Marella et al., 2013). More peptide transporters than amino acid transporters were induced by CN (Puthoff et al., 2003). Most of the investigated amino acid transporters were expressed in galls suggesting a role for these transporters in the amino acid transport ability of infected roots (Fischer et al., 1995; Okumoto et al., 2002). Amino acid transporters of the amino acid permease (AAP) family were reported to be induced in syncytia (Szakasits et al., 2009; Hofmann et al., 2009). Also, amino acid transporters, like *AAP3* and *AAP6* were demonstrated to play a role during *Arabidopsis*-RKN interaction. RKN infection on *AtAAP3* and *AtAAP6* knock-out plants was significantly reduced in comparison with wild-type (Marella et al., 2013). Two putative auxin transporter genes of the AAAP (amino acid auxin permeases) superfamily, *AtAUX1* and *AtAUX4/LAX3,* were shown to be expressed in syncytia (Hammes et al., 2005). Beside of auxin that is essential for syncytia development (Viglierchio and Yu, 1968; Goverse et al., 2000), other phytohormones such as cytokinin, ethylene, *CLAVATA* elements, CEP (C-terminally encoded peptide), and PSK (phytosulfokines) are also associated with the RKN or CN feeding cell formation (e.g., Goverse and Bird, 2011; Rodiuc et al., 2012). As well, the amino acid transporter, AtCAT6 is induced in giant cells similarly to other amino acid sink plant tissues, however, it is not essential for the establishment of RKN feeding sites (Hammes et al., 2006). This might be due to redundancy in transporter expression or compensatory expression of other transporters in *AtCAT6* knockout plants. In addition, nutrient loading into giant cells depends not only on the apoplastic step but also occurs symplasticallybrea via PD.

Water import into feeding sites seems to involve genes responsible for water transport (Grundler and Hofmann, 2011). Considering that the plasma membrane has a restricted ability for water transport and diffusion, this process can be assisted by aquaporins forming water pores along the plasmalemma (Johansson et al., 2000). Aquaporins in plants are observed within the plasma membrane and in the tonoplast (vacuolar membrane; Maurel et al., 1993; Kammerloher et al., 1994). Plasma membrane aquaporins and homologs are named PIPs (plasma membrane intrinsic proteins) and tonoplast aquaporins and homologs are called tonoplast intrinsic proteins (TIPs). Elevated expression of the aquaporins *TobRB7, AtPIP2.6 AtPIP2.5* has been observed by microarray of *Meloidogyne incognita*-infected roots (Opperman et al., 1994; Hammes et al., 2005). Localization of *AtPIP2*.*5* in galls suggests that this gene might be a functional ortholog of a giant-cell-specific aquaporin *TobRB7* of tobacco (Opperman et al., 1994; Puthoff et al., 2003). *AtPIP2.5* was also induced upon infection with the beet CN, *H. schachtii* (Puthoff et al., 2003). Upregulation of both types of aquaporins (PIPs and TIPs) in galls has been proposed to be associated with a volume increase of giant cells (Barcala et al., 2010). The developing nematode is continually ingesting the giant cell contents in order to rapidly grow and reproduce.

## CONCLUSIONS AND PERSPECTIVES

Plant parasitic nematodes are reliant on water and nutrient resources from their host plants. Feeding strategies applied by plant-parasitic nematodes will determine how efficient the supply of solutes will be. These strategies greatly depend on sophisticated cell wall modifications of the feeding site in order to transform vascular parenchymatic cells into TCs. These cellular adaptations are of key relevance in order that sedentary endoparasitic nematodes capture enough water and nutrient supply for their development and reproduction.

Nematode-induced syncytia or giant cells constitute a multinucleate model of TCs, and it is generally believed that wall protuberances arise as a result of nematode demand for nutrients. Although some information on solute supply has been mostly reported for syncytia induced by *H. schachtii* in *Arabidopsis*, many questions remain open. Currently it is not yet comprehensible what signals are employed to cause the establishment of these sink structures (galls and syncytia) and the transport occurring within these solute transfer sites involving the feeding cell and numerous phloem and xylem elements along this sink. It remains ambiguous as to what switches ordinary plant cells into transfer-feeding cells that supply nematodes with solutes for their development and reproduction. These plant cellular morphological changes might be caused directly or indirectly by secreted products or feeding activity employed by the nematode during parasitism. What triggers and regulates the switch from apoplasmic to symplastic solute supply and the gating of PD is yet to be discovered. Further functional genetic approaches as well as microscopic studies will help to elucidate genes involved in this process in order to comprehend how nematode induced TCs operate.

## Conflict of Interest Statement

The authors declare that the research was conducted in the absence of any commercial or financial relationships that could be construed as a potential conflict of interest.
